# Isolation and Characterization of Two Persimmon Xyloglucan Endotransglycosylase/Hydrolase (*XTH*) Genes That Have Divergent Functions in Cell Wall Modification and Fruit Postharvest Softening

**DOI:** 10.3389/fpls.2016.00624

**Published:** 2016-05-11

**Authors:** Ye Han, Qiuyan Ban, Yali Hou, Kun Meng, Jiangtao Suo, Jingping Rao

**Affiliations:** State Key Laboratory of Crop Stress Biology for Arid Areas, College of Horticulture, Northwest A&F UniversityYangling, China

**Keywords:** Xyloglucan endotransglycosylase/hydrolase, Fruit softening, XET, Persimmon, Cell wall

## Abstract

Fruit cell wall modification is the primary factor affecting fruit softening. Xyloglucan endotransglycosylase/hydrolase (XTH), a cell wall-modifying enzyme, is involved in fruit softening. In this study, two novel *XTH* genes (*DkXTH6* and *DkXTH7*) were identified from persimmon fruit. Transcriptional profiles of both of the two genes were analyzed in different tissues of persimmon, and in response to multiple hormonal and environmental treatments [gibberellic acid (GA_3_), abscisic acid (ABA), propylene, and low temperature]. Expression of *DkXTH6* was positively up-regulated during ethylene production and by propylene and ABA treatments, and suppressed by GA_3_ and cold treatment. In contrast, *DkXTH7* exhibited its highest transcript levels in GA_3_-treated fruit and cold-treated fruit, which had higher fruit firmness. We found that DkXTH6 protein was localized in cell wall by its signal peptide, while cytoplasmic DkXTH7 protein contained no signal peptide. When expressed *in vitro*, the recombinant proteins of both DkXTH6 and DkXTH7 exhibited strict xyloglucan endotransglycosylase (XET) activity but no xyloglucan endohydrolase (XEH) activity. The recombinant protein of DkXTH6 showed a higher affinity with small acceptor molecules than the recombinant DkXTH7. Taken together with their opposing expression patterns and subcellular localizations, these results suggested that DkXTH6 might take part in cell wall restructuring and DkXTH7 was likely to be involved in cell wall assembly, indicating their special roles in persimmon fruit softening.

## Introduction

Persimmon (*Diospyros kaki* L. cv Fuping jianshi) is rich in nutrition and has a unique flavor, but it softens and decays quickly, which affects badly its marketability (Zhang et al., 2012; Lv et al., 2014). Fleshy fruit softening is associated with significant biochemical changes in cell wall fractions (Vicente et al., 2007; Matas et al., 2009), usually resulting from cell wall polymer breakdown catalyzed by various cell wall enzymes, such as polygalacturonase, pectate lyase, β-galactosidase, cellulase, and xyloglucan endotransglycosylase/hydrolase (XTH; Cosgrove, 2005; Figueroa et al., 2008; Payasi et al., 2009). It has been estimated that pectic and hemicellulosic polysaccharides are the predominant components undergoing depolymerization and solubilization during fruit softening (Brummell and Harpster, 2001).

In the cell wall of most dicotyledons, xyloglucan is the major hemicellulosic polysaccharide, which can form skeletal networks with the cellulose fibrils to confer strength and rigidity on the wall (Schroder et al., 1998; Zhu et al., 2013). XTH, an important enzyme involved in xyloglucan metabolism, can function as a xyloglucan endotransglycosylase (XET) and/or a xyloglucan endohydrolase (XEH), with the former transferring one xyloglucan molecule fragment to another and the latter responsible for hydrolysis of one xyloglucan molecule (Nishitani, 1997; Rose et al., 2002; Eklof and Brumer, 2010). Moreover, it has been demonstrated that there are two types of endotransglycosylase; integrational XET catalyzes a newly secreted xyloglucan molecule's reaction with a previously formed wall-bound xyloglucan, while restructuring XET catalyzes a reaction between two preformed wall-bound xyloglucan molecules (Thompson and Fry, 2001).

XTHs were previously thought to be responsible for fruit softening and textural changes during storage by breaking down the cellulose-xyloglucan matrix and loosening the cell wall, as has been described in fruits, such as apple (Munoz-Bertomeu et al., 2013), longan (Feng et al., 2008; Zhong et al., 2008), grape berry (Ishimaru and Kobayashi, 2002), lychee (Lu et al., 2006), and tomatoes (Saladie et al., 2006; Miedes and Lorences, 2009). However, some authors reported that XET activity could be associated with the maintenance of structural integrity of the cell wall rather than for dismantling it (Hiwasa et al., 2004; Fonseca et al., 2005; Nishiyama et al., 2007; Miedes et al., 2010). In transgenic tomatoes, Miedes et al. (2010) suggested that the XET activity was involved in maintaining the structure of cell wall and that its decrease during fruit ripening could contribute to fruit softening. Meanwhile, XET activity exhibited high levels in some rapidly growing tissues and were involved in plant cell expansion (Nishitani and Tominaga, 1992; Thompson et al., 1997; Atkinson et al., 2009).

Persimmon fruits not only have a unique flavor but are also ideal for the purpose of studying softening because of evident changes in texture during ripening. The *XTH* gene family members each play a certain role in plant growth, fruit ripening, and fruit softening (Sulova et al., 2003; Eklof and Brumer, 2010). To date, five *XTH* genes (*DkXTH1*-*5*) were cloned from persimmon, and their roles in fruit growth and ripening have been discussed (Han et al., 2015). However, more *XTH* genes should be studied based on their special roles. Hence, in this study, we isolated two novel *XTH* genes in persimmon (*DkXTH6* and *DkXTH7*). Their expression patterns were analyzed in different tissues and in persimmon fruits under various treatments that yielded differing postharvest softening rates. Furthermore, the subcellular localization of *DkXTH* genes was assessed, and the enzymatic characteristics of recombinant isoenzymes were also studied to explore their divergent functions in cell wall modification and persimmon fruit softening.

## Materials and methods

### Plant materials and treatments

Persimmon material (*Diospyros kaki* L. cv Fuping jianshi) was obtained from a commercial orchard in Fuping County, Shaanxi Province, China. Materials were transported to the laboratory within 3 h after harvest. Flowers and stems were picked at anthesis. Young leaves were picked while rapidly expanding (at ~4 × 6 cm in size), whereas ripe leaves were picked when fully expanded (~10 × 15 cm). Young fruits were picked at 40 days after full bloom, whereas ripe fruits were picked at 150 days after full bloom.

For postharvest softening and senescence analysis, fruits of uniform size and without visible defects were harvested with 70–80% surface yellow coloration. The selected fruits were divided randomly into five experimental groups, with 180 fruits in each group. The first and second groups were immersed for 2 min into 60 mg L^−1^ GA_3_ (“GA_3_”; G7645; Sigma-Aldrich, St. Louis, MO, USA) or 50 mg L^−1^ ABA (“ABA”; A1049; Sigma-Aldrich) respectively. The third group was placed in a 360 L chamber and exposed to 5000 μL L^−1^ propylene for 24 h (“propylene”), and the fourth group without any treatment served as the control (“CK”). The first four groups were stored at 25 ± 1°C, while the fifth group was stored at 0 ± 1°C (“cold”). After treatment, the fruits of each group were divided randomly into three subgroups, and samples were chosen randomly from subgroups every 4 days for the determination of ethylene production, respiration rate, and firmness. All of the tissue samples were frozen immediately in liquid nitrogen and stored at −80°C until use.

### Fruit firmness, respiration rate, and ethylene production

Fruit firmness was measured at three locations at 120° intervals around the equatorial axis of the fruit after removing a small disk of skin. A pressure tester (model FT327; Effegi, Milan, Italy) was used equipped with a 5 mm diameter probe. For each time point, six fruits were used for replications.

Six fruits from each treatment subgroup were enclosed in a 9.17 L vessel and sealed at storage temperature for 1 h, after which 1 ml of gas was collected by a syringe three times. Ethylene production was determined by injecting a gas sample into a flame ionization detection GC-14A gas chromatograph (Shimadzu, Kyoto, Japan), as described by Zhu et al. (2013). The respiration rate was measured by a CO_2_ infrared gas analyzer (TEL7001; GE Telaire, CA, USA), according to Han et al. (2015).

### RNA extraction and *DkXTHs* cloning

Total RNA was isolated from the frozen tissues using the hot borate method (Wan and Wilkins, 1994). First strand cDNA was synthesized using a PrimeScript RT Reagent Kit with gDNA Eraser (TaKaRa, Dalian, Japan), according to the manufacturer's instructions. Subsequently, the conserved regions of persimmon *XTH* genes were isolated using degenerate primers designed previously (Zhu et al., 2013). To obtain the full-length open reading frame (ORF) of *XTH* genes, 3′- or 5′-rapid amplification of cDNA ends (RACE) polymerase chain reactions (PCR) were performed using RACE cDNA amplification kits (TaKaRa), according to the manufacturer's protocol. All PCR fragments were purified and inserted into the pMD18-T vector (TaKaRa) and sequenced by GenScript, Inc. (Nanjing, China). The primer sequences are listed in Table 1.

**Table 1:** **Oligonucleotide sequences for primers used in this study**.

**Gene name**	**Gene bank accession number**	**Prime sequences (5′–3′)**	**Purpose**
*DkXTH6*	KC511053	Outer: ACTCCCATTGGCTGGTCCTT	*DkXTH6* 5′RACE
		Inner: GTTCCAGAGGAGGGAGTAAGAGT	
		Outer: ATGGAACGCCGACGATTGGG	*DkXTH6* 3′RACE
		F: AGTTGTTTCAGCCGAGTTGGG	*DkXTH6* full-length cDNA clone
		R: ACCTAGTGGCGGTGGTGTTC	
		F: GGGCAAGTATTTGTTCGG	*DkXTH6* RT-qPCR
		R: CCAGAGGAGGGAGTAAGAG	
		F:GCTCTAGAATGGCTTCTTCTCTAACTC	DkXTH6Full
		R:GGGGTACCGTGGCGGTGGTGTTCGCACT	
		F:GCTCTAGAATGGCTTCTTCTCTAACTC	DkXTH6sp
		R:GGGGTACCACCCCCCATTGCAGAAGCAA	
		F:GCTCTAGATCGATGAATTCGTCCCGATT	DkXTH6Int
		R:GGGGTACCGTGGCGGTGGTGTTCGCACT	
		F: CGGGATCCTCGATGAATTCGTCCCGATTC	*DkXTH6* recombinant protein expression
		R:CCCAAGCTTGTGGCGGTGGTGTTCGCACT	
*DkXTH7*	KC541541	Outer: CAGTCATCGGCATTCCACAT	*DkXTH7* 5′RACE
		Inner: CTTTGCCTTGGCTAAACACG	
		Outer:: TGGCAACTTACTATCTGTCTTCG	*DkXTH7* 3′RACE
		F: CGTGGACACCTTCGTTTCTC	*DkXTH7* full-length cDNA clone
		R: CGCATCTTGTCCACGCAAT	
		F: AGGCAAAGGCAATAGGG	*DkXTH7* RT-qPCR
		R: TCATCGGCATTCCACAT	
		F:GCTCTAGAATGAACGCCGAAGGCGGAAA	DkXTH7Full
		R:GGGGTACCAGAAATGTTGCATTCTGGAGCG	
		F:CGGGATCCATGAACGCCGAAGGCGGAAA	*DkXTH7* recombinant protein expression
		R:CCCAAGCTTAGAAATGTTGCATTCTGGAGCG	
Actin	AB219402	F:TGCTCTTCCAGCCATCACTCATT	*Actin* RT-qPCR
		R:ATTTCCTTGCTCATCCGGTCAG	

*Letters “F” and “R” indicate the forward and reverse primers, respectively*.

### Sequence analysis and bioinformatic methods

The BLAST program in GenBank (http://blast.ncbi.nlm.nih.gov/Blast.cgi) was used to confirm the nucleotide sequences that were obtained by RT-PCR clone. ORF detection and amino acid sequence deduction were performed according to NCBI ORF Finder (http://www.ncbi.nlm.nih.gov/gorf/gorf.html). The alignment and comparison of the deduced amino acid sequences were conducted using the DNAMAN program. Candidate protein sequences of various physical and chemical parameters, including molecular weight and theoretical isoelectric point (pI), were calculated using the PeptideMass program (http://us.expasy.org/tools/peptidemass.html). SignalP (http://www.cbs.dtu.dk/services/SignalP/) was used to analyze the N-terminal signal peptide of the putative protein. The phylogenetic tree was generated based on the Neighbor-Joining method by using 1000 bootstrap replicates followed by MEGA 5.1 software. The three-dimensional structures of DKXTH proteins were predicted, and tertiary structures were modeled using the Swiss-Model workspace (http://swissmodel.expasy.org).

### Expression analysis by RT-qPCR

The first-strand cDNA was synthesized according to the methods described above. Quantitative real-time (qRT)-PCR (20 μL total volume) was performed using 1.0 μL cDNA (300 ng), 7.4 μL ddH_2_O, 0.8 μL of each primer (10 μmol L^−1^), and 10 μL SYBR Premix Ex TaqTMII (TaKaRa) using an iCycler iQ5 (Bio-Rad, Hercules, CA, USA). The cycling conditions included an initial hot start at 95°C for 3 min, followed by 40 three-step cycles of 95 °C for 10 s, 55°C for 30 s, and 72°C for 20 s. Expression of the persimmon *ACTIN* gene was used to normalize the mRNA levels, and no-template controls for each primer pair were included in each run. Serial dilutions of cDNA were used to calibrate a standard curve for each gene to ensure the minimal resultant efficiencies between actin primers and gene-specific primers. The gene relative expression level was calculated through the comparative C_*T*_ (2^−ΔΔCT^) method (Livak and Schmittgen, 2001), and the expression levels at the harvest time point were set to 1. All of the samples had three biological replicates, and the specific primer sequences used for qRT-PCR are listed in Table 1.

### Subcellular localization

The ORF of *DkXTH6/7* sequence (DkXTH6/7Full), the signal peptide sequence of *DkXTH6* (DkXTH6sp), and the ORF sequence of *DkXTH6* without signal peptide (DkXTH6Int) were isolated using the specific primers listed in Table 1. Four confirmed sequences were cut with *Xho*I and *Kpn*I restriction enzymes and then inserted into the pBI 221-GFP vector, which contained the green fluorescent protein (*GFP*) gene after the multiple clone site. Onion epidermal cells were bombarded with the four divergent recombinant plasmids (5 μg) using a biolistic PDS-1000/He particle delivery system (Bio-Rad). After being cultivated on Murashige and Skoog media for 24 h in the dark (22°C), the onion epidermal cells were examined and imaged by a confocal laser-scanning microscope (A1R; Nikon, Tokyo, Japan).

### Production and purification of recombinant XTH proteins and enzyme activity analysis

The ORFs of *DkXTH6* and *DkXTH7* without the signal peptide sequence were amplified by PCR with the combinations of specific primers (Table 1). The resulting PCR products were digested with the corresponding restriction enzymes (underlined in the primers) and ligated into *Bam*HI- and *Hind*III-digested pET-32a vector. Heat-shock transformed *Escherichia coli* BL21 was grown in Luria-Bertani-rich medium, and the production of recombinant proteins was induced with 0.5 mM isopropyl β-d-thiogalactopyranoside. The bacterial cells were then treated by sonication, and the crude proteins were harvested by centrifugation at 15, 000 × g for 10 min. The pellet was dissolved in binding-wash buffer (8 M urea, 40 mM Tris-HCl, 0.5 M NaCl, 20 mM imidazole, 10% glycerol, pH 7.9), then purified by nickel-nitrilotriacetic acid (Ni-NTA) resin column (DP101; TransGen Biotech, Beijing, China) according to the manufacturer's instructions. Subsequently, purified recombinant proteins were refolded in a linear urea buffer containing 2 mM GSH/0.2 mM GSSG, 0.3 M l-arginine, 10% glycerol, 0.5 M NaCl, 1 mM EDTA, and 40 mM Tris-HCl, pH 7.9. The empty vectors were taken as blank control. The purified recombinant proteins of *DkXTH6* and *DkXTH7* are referred to as DkXTH6-RP and DkXTH7-RP, respectively.

A small volume of the purified protein was used for SDS-polyacrylamide gel electrophoresis stained with Coomassie Brilliant Blue R-250, and the rest of the target protein was concentrated and dialyzed in a citrate/phosphate buffer, pH 5.5, to determine XET/XEH activity as described in Han et al. (2015). Briefly, XET activity was measured by a colorimetric assay using xyloglucan oligosaccharides (XGOs) expressed in arbitrary units. Moreover, a viscometric assay was used to measure the XEH activity by depolymerizing xyloglucan, and *Trichoderma reesei* cellulase (Sigma-Aldrich) was used as the control enzyme. The pH rate profile of proteins was analyzed over a pH range of 3–8, and the dependence of relative XET activity of proteins on the concentration of added XGOs was measured using XGOs ranging from 0.01 to 0.20 mg mL^−1^.

### Statistical analysis

Data were measured by analysis of variance using SPSS, version 22.0, and the means were compared by Fisher's least significant difference test. *P* values below 0.05 were considered statistically significant (*P* < 0.05). All measured data are presented as mean ± standard error of the means.

## Results

### Cloning and phylogenetic analysis of *DkXTH6* and *DkXTH7*

Two novel full-length sequences designated as *DkXTH6* and *DkXTH7* were isolated from persimmon fruit and submitted to GenBank with the accession numbers of KC511053 and KC541541, respectively. The *DkXTH6* cDNA revealed a complete ORF spanning between 56 and 900 base pair (bp) positions, encoding a predicted polypeptide of 299 amino acid residues and corresponding to a calculated molecular mass of 33.76 kDa and a theoretical pI of 5.48. The *DkXTH7* cDNA, 1105 bp, consisted a full-length ORF of 807 bp (106–912 bp), and a deduced polypeptide of nearly 30.83 kDa, which comprised of 268 amino acids with a pI of 6.32. The deduced peptide sequence of DkXTH6 shared 53.31% amino acid homology with that of DkXTH7. Furthermore, the signal peptide sequence of DkXTH6 was predicted, and the cleavage site was between 20 and 21 amino acids. However, the encoded protein of DkXTH7 was predicted to contain no signal peptides.

**Figure 1:**
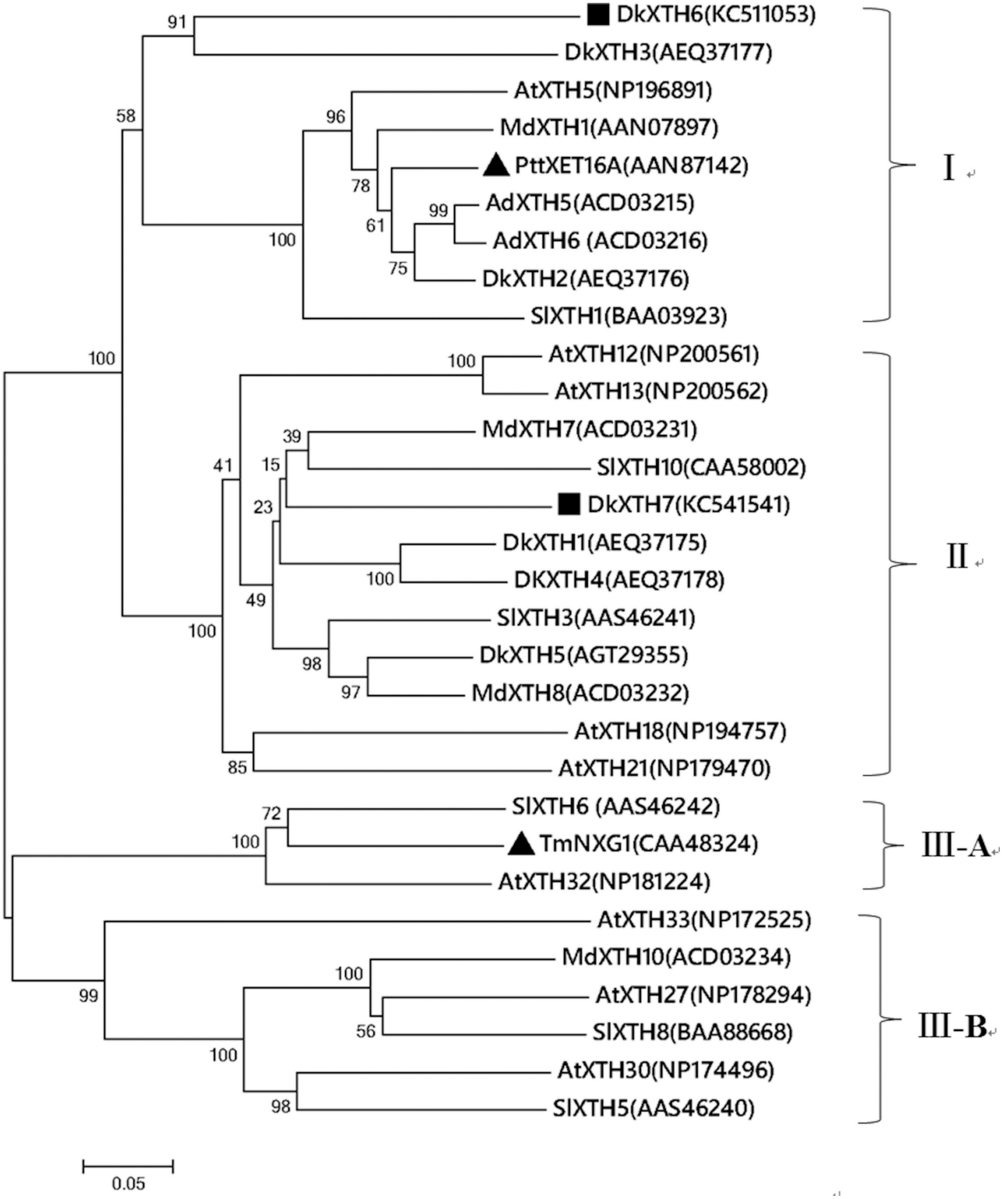
**Phylogenetic tree of the deduced amino acid sequences of XTHs**. The phylogenetic tree was constructed by the Neighbour-Joining method (1000 trials) with bootstrap using MEGA 5.1 software. The distance scale length of the tree was 0.05 and the bootstrap values were indicated above the branch. DkXTH6 and DkXTH7 are set as bold (square). PttXET16A and TmNXG1 (triangle) were the first XET and XEH with three-dimensional structures, respectively. The GenBank accession numbers are indicated in the figure.

To ascertain the evolutionary relationship of persimmon *DkXTH6* and *DkXTH7* genes among other plant species, a phylogenetic tree on the amino acid level was constructed (Figure 1). Results showed that 30 plant *XTHs* could be divided into three groups; group III was divided into subgroup III-A and III-B, as reported previously (Campbell and Braam, 1999). Group I included DkXTH2, DkXTH3, and DkXTH6, which was grouped together with PttXET16A, the first XET with a three-dimensional structure have been reported (Johansson et al., 2004). Meanwhile, DkXTH7 belonged to group II and is closely related to the tomato protein SlXTH10 and the apple protein MdXTH7, showing similarities of 59.2 and 63.7%, respectively. In addition, TmNXG1, the first XEH with a three-dimensional structure (Baumann et al., 2007), was classified into subgroup III-A.

**Figure 2:**
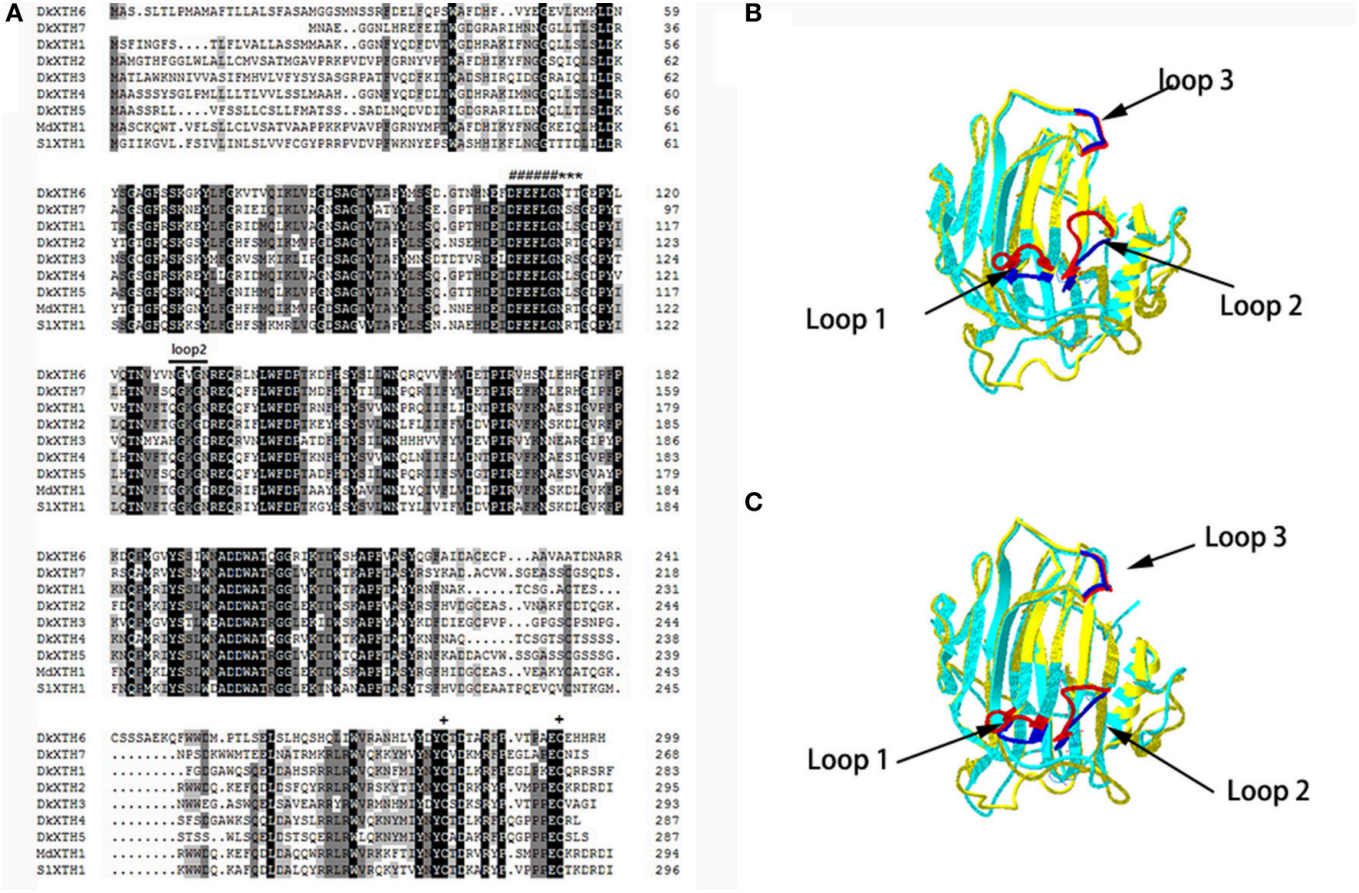
**Alignment of predicted DkXTHs proteins and the prediction of three-Dimensional structures of DkXTH6 and DkXTH7**. **(A)** Black shading represents identical amino acids, and gray shading identifies the residues shared by at least three of the XTHs. Putative catalytic domain, N-glycosylation site, and two cysteines are marked with “#,” “^*^,” and “+,” respectively. Straight lines identify loops 2 of DkXTH6 and DkXTH7. **(B)** The predicted three-dimensional structures of DkXTH6 and DkXTH7 based on the template of the crystal structure of PttXET16A using Swiss-Model workspace. Superimposition of the structures of DkXTH6 (yellow + blue) and TmNXG1 (light blue + red) highlighting the different conformations of three loops. **(C)** Superimposition of the structures of DkXTH7 (yellow + blue) and TmNXG1 (light blue + red) highlighting the different conformations of three loops. In TmNXG1, loop 1 was from Asn-84 to Asp-93; loop 2 was from Glu-117 to Gly-126; and loop 3 was from Trp-190 to Tyr-197.

A multiple alignment of the putative DkXTH1-7 with other plant XTH homologs was performed to determine their relatedness (Figure 2A). DkXTH6 and DkXTH7 possessed several functional domains typical in plant XTHs, including the conserved amino acids (DEIDFEFLG) as a putative active site and together with a potential N-linked glycosylation (N-X-S/T) site. In addition, both DkXTH6 and DkXTH7 contained two cysteine residues in the carboxyl-terminal region. Compared with the strict XET enzymes, XEH enzymes show three evidently different loops in three-dimensional structures; the length of loop 2 might have an important role in balancing XET and XEH activity (Baumann et al., 2007; Eklof and Brumer, 2010). The three-dimensional structures of DkXTH6 and DkXTH7 were first elucidated by homology modeling based on the X-ray structure of the PttXET16A protein (Protein Data Bank code 1un1), which displayed high-sequence identity with persimmon XTHs. Both in DkXTH6 and DkXTH7, loop 2 contained five amino acids (Asn-128 to Asn-132 in DkXTH6; Gln-103 to Asn-107 in DkXTH7; Figure 2A), the same number of amino acids as found in the PttXET16A, but they all had less amino acids than in the TmNXG1 loop 2, which had 10 amino acids (Figures 2B,C).

### Physiological characterization during persimmon fruit storage

**Figure 3:**
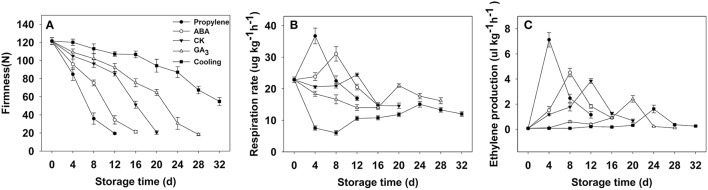
**Firmness (A), respiration rate (B) and ethylene production (C) of persimmon fruits during storage**. “propylene” “ABA,” and “GA_3_” indicated Fuping Jianshi fruit treated with propylene (5000 μl L^−1^, 24 h), ABA (50 mg L^−1^, 2 min), and GA_3_(60 mg L^−1^, 2 min), respectively, and stored at 25°C. The fruit without any treatment and stored at 25 and 0°C was served as the “CK” and “cold,” respectively. The vertical bars indicate the standard errors of three biological replicate assays.

Fruit firmness was recorded for all postharvest samples at 4-day intervals starting from the day after harvest. The firmness of CK fruit (“Fuping jianshi” fruit without any treatment stored at 25°C) showed an obvious decrease at 12 days after harvest and decreased from 121.5 to 20.7 N on day 20 (Figure 3A), whereas the propylene and ABA fruit (Fuping jianshi fruit treated with propylene and ABA, respectively, and stored at 25°C) exhibited a higher rate of softening. When tested for firmness, CK fruit was 19 and 77% more firm than propylene fruit at 4 and 12 days of storage, respectively. Meanwhile, CK fruit was 22 and 58% more firm than ABA fruit at 8 and 16 days of storage, respectively. By contrast, the GA_3_ fruit (Fuping jianshi' fruit treated with GA_3_ and stored at 25°C), and cooling fruit (Fuping jianshi fruit without any treatment stored at 0°C) showed a strong suppression of softening, which were 68 and 78% firmer than CK fruit at 20 days of storage, respectively.

Respiration rate was stimulated by application of propylene and ABA, and suppressed by GA_3_ and low temperature (Figure 3B). The maximal respiration rate in propylene fruit (4 days) and ABA fruit (8 days) was 33 and 21% higher than that in CK fruit (12 days), respectively, whereas the maximal respiration rate in GA_3_ fruit (20 days) and cooling fruit (24 days) was only 86 and 62% of that in CK fruit (12 days), respectively.

All of the treated fruits exhibited a typical climacteric ethylene production pattern during storage (Figure 3C). The maximal ethylene production in propylene fruit (4 days) and ABA fruit (8 days) was 46 and 15% higher than that of CK fruit (12 days), respectively, showing the acceleration in ethylene biosynthesis by propylene and ABA. By contrast, the maximal ethylene production in GA_3_ fruit (20 days) and cooling fruit (24 days) was only 63 and 42% of that in CK fruit (12 days), respectively, suggesting that ethylene production was strongly inhibited by GA_3_ and low temperature.

### Expression of persimmon *DkXTHs* in different tissues

**Figure 4:**
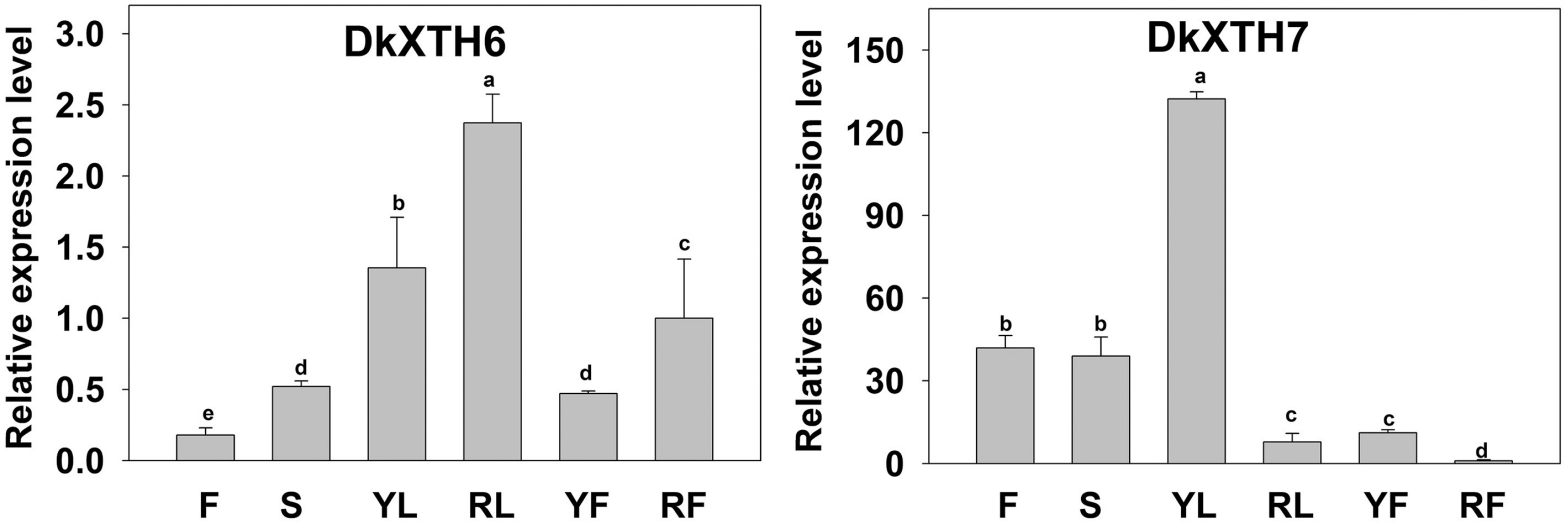
**Expression pattern of *DkXTH6* and *DkXTH7* in various tissues of persimmon fruits**. The *DkXTH* mRNA levels are relative to those of *Actin* mRNA. “F” and “S” are indicated the flowers and stems picked at anthesis, respectively. Young leaves (“YL”) were picked while rapidly expanding (at ~4 × 6 cm in size), whereas ripe leaves (“RL”) were picked when fully expanded (~10 × 15 cm). Young fruits (“YF”) were picked at 40 days after full bloom, whereas ripe fruits (“RF”) were picked at 150 days after full bloom. Expression of gene at “RF” was used as the control with a nominal value of 1. Vertical bars indicate the standard error of three replicate assays. Columns with different letters at each time point are significantly different (LSD, *P* = 0.05).

A quantitative RT-PCR analysis was performed to reveal the expression pattern of persimmon *DkXTH6* and *DkXTH7* genes in various tissues (Figure 4). The transcripts of the two genes could be detected in all of the tested tissues, including leaf, stem, flower, and fruit. Interestingly, the expression level of *DkXTH6* in mature tissues was evidently higher than that in fast growing tissues. By contrast, the *DkXTH7* mRNA was expressed at an extremely high level in both leaf and fruit fast growing tissues.

### Expression of *DkXTHs* during persimmon fruit storage

**Figure 5:**
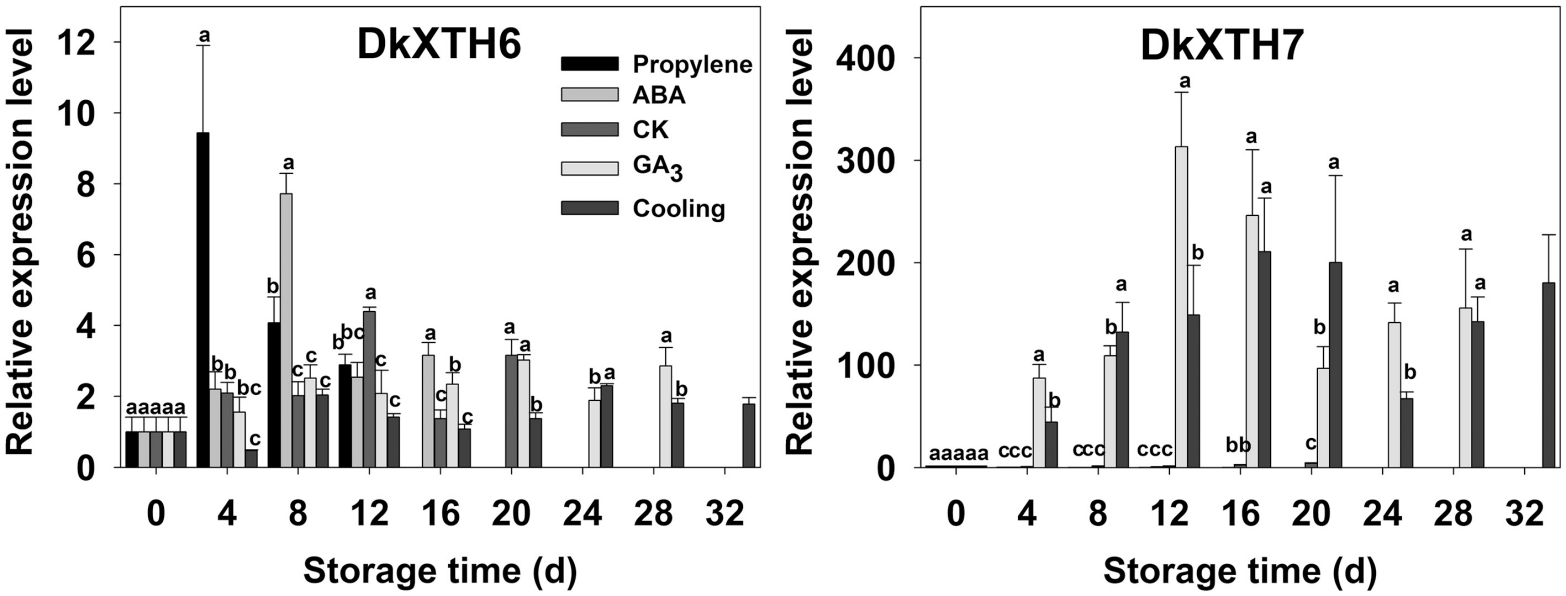
**Expression pattern of *DkXTH6* and *DkXTH7* in persimmon fruits during storage**. The *DkXTH* mRNA levels are relative to those of *Actin* mRNA. “propylene,” “ABA,” and “GA_3_” indicated Fuping Jianshi fruit treated with propylene (5000 μl L^−1^, 24 h), ABA (50 mg L^−1^, 2 min), and GA_3_(60 mg L^−1^, 2 min), respectively, and stored at 25°C. The fruit without any treatment and stored at 25 and 0°C was served as the “CK” and “cold,” respectively. The vertical bars indicate the standard errors of three biological replicate assays. Expression of gene at 0 d was used as the control with a nominal value of 1. Columns with different letters at each time point are significantly different (LSD, *P* = 0.05).

After harvest, the relationships of *DkXTH6* and *DkXTH7* genes with softening were addressed in propylene, ABA, CK, GA_3_, and cooling fruit by monitoring changes in transcript levels using real-time quantitative PCR (Figure 5, Supplementary Tables 1, 2). In CK fruit, expression level of *DkXTH6* increased rapidly and peaked on the same day (12 days) as ethylene production, then dramatically decreased. The expression level seemed parallel to the pattern of ethylene production, maintaining high levels during the quick declining fruit firmness. In propylene and ABA fruit, *DkXTH6* exhibited the similar expression pattern but with higher maximal values than that in CK fruit. In detail, the maximal expression levels of *DkXTH6* were 53.4 and 43.0% higher in stored propylene and ABA fruit than in CK fruit, showing the synergistic effect of propylene and ABA on *DkXTH6* expression. GA_3_ and cooling fruit exhibited lower expression levels of *DkXTH6*, with respective maximal values of only 68.8 and 52.5% of that in CK fruit.

By contrast, the expression level of *DkXTH7* was evidently higher in GA_3_ and cooling fruit, and the maximal values were 76.1 and 51.2 fold higher than the expression level in CK fruit, showing that GA_3_ and low-temperature treatment significantly induced *DkXTH7* gene expression. After harvest, expression level of *DkXTH7* increased rapidly in both GA_3_ fruit and cooling fruit; however, the peak of ethylene production was accompanied with lower expression levels, and the fruit firmness declined quickly.

### Subcellular localization

**Figure 6:**
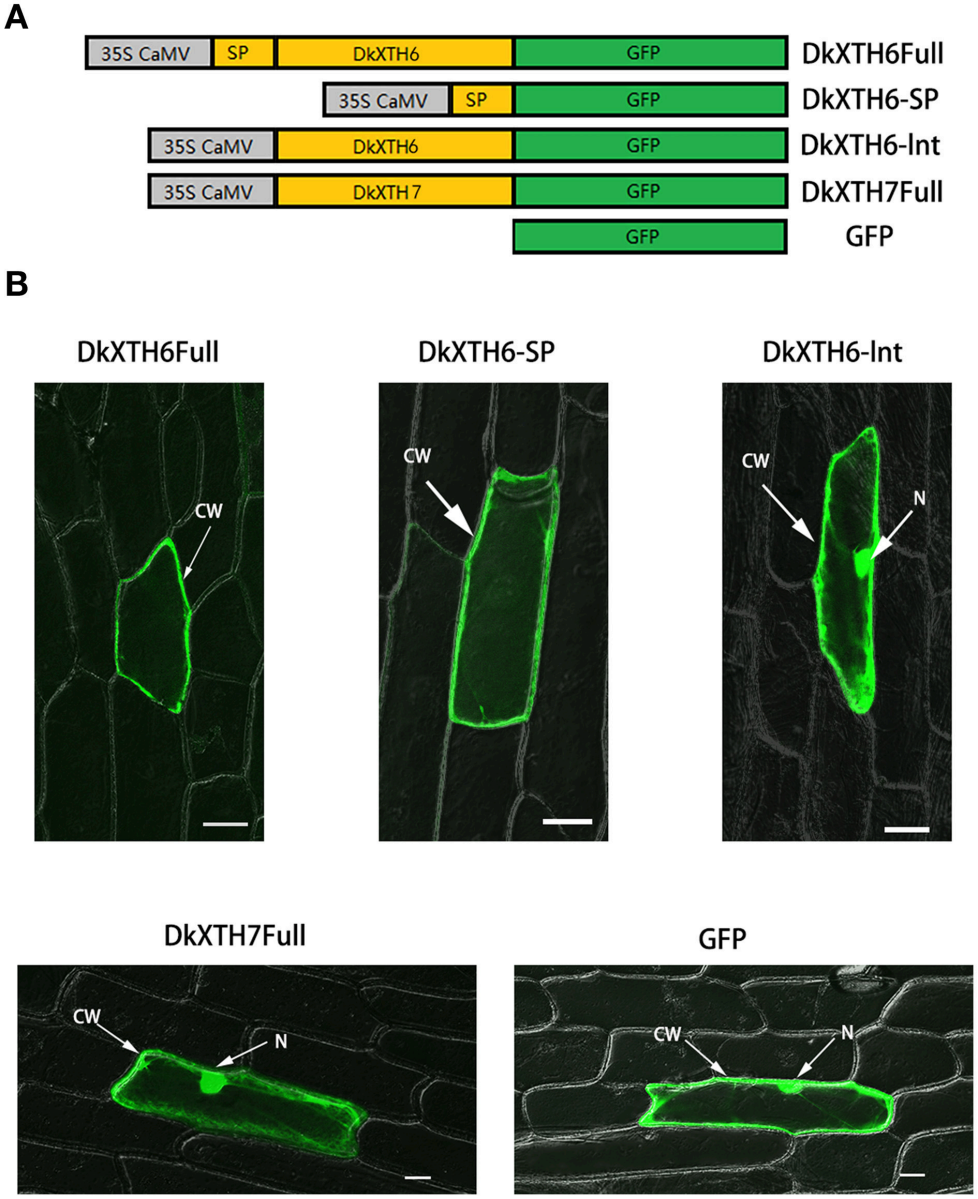
**Subcellular localization of DkXTH6 and DkXTH7 by transient expression in onion epidermal cells**. **(A)** Diagram of *DkXTH6* and *DkXTH7* constructs fused to *GFP*. **(B)** Panels 1, 2, and 3, transmission and fluorescence images of subcellular localization of *DkXTH6*; panels 4 and 5, transmission and fluorescence images of subcellular localization of *DkXTH7* and *GFP* control, respectively. CW, cell wall; N, nucleus.

To investigate the subcellular localization of DkXTH6 and DkXTH7 proteins, onion epidermal cells were bombarded with four divergent recombinant plasmids named DkXTH6Full, DkXTH6sp, DkXTH6Int, and DkXTH7Full (Figure 6A). As shown in Figure 6B, both DkXTH6Full and DkXTH6sp were located in cell wall, however, the GFP control was located in the whole cells. Meanwhile, DkXTH6Int, in which there was an absence of the signal peptide, was located throughout the cells. Similarly, DkXTH7Full, which was predicted to contain no signal peptide, was dispersed throughout the cells. These results indicated that the coding proteins of DkXTHs, which contained signal peptides, may target the cell wall by their N-terminal signal peptides.

### Recombinant XTH protein expression and activity

**Figure 7:**
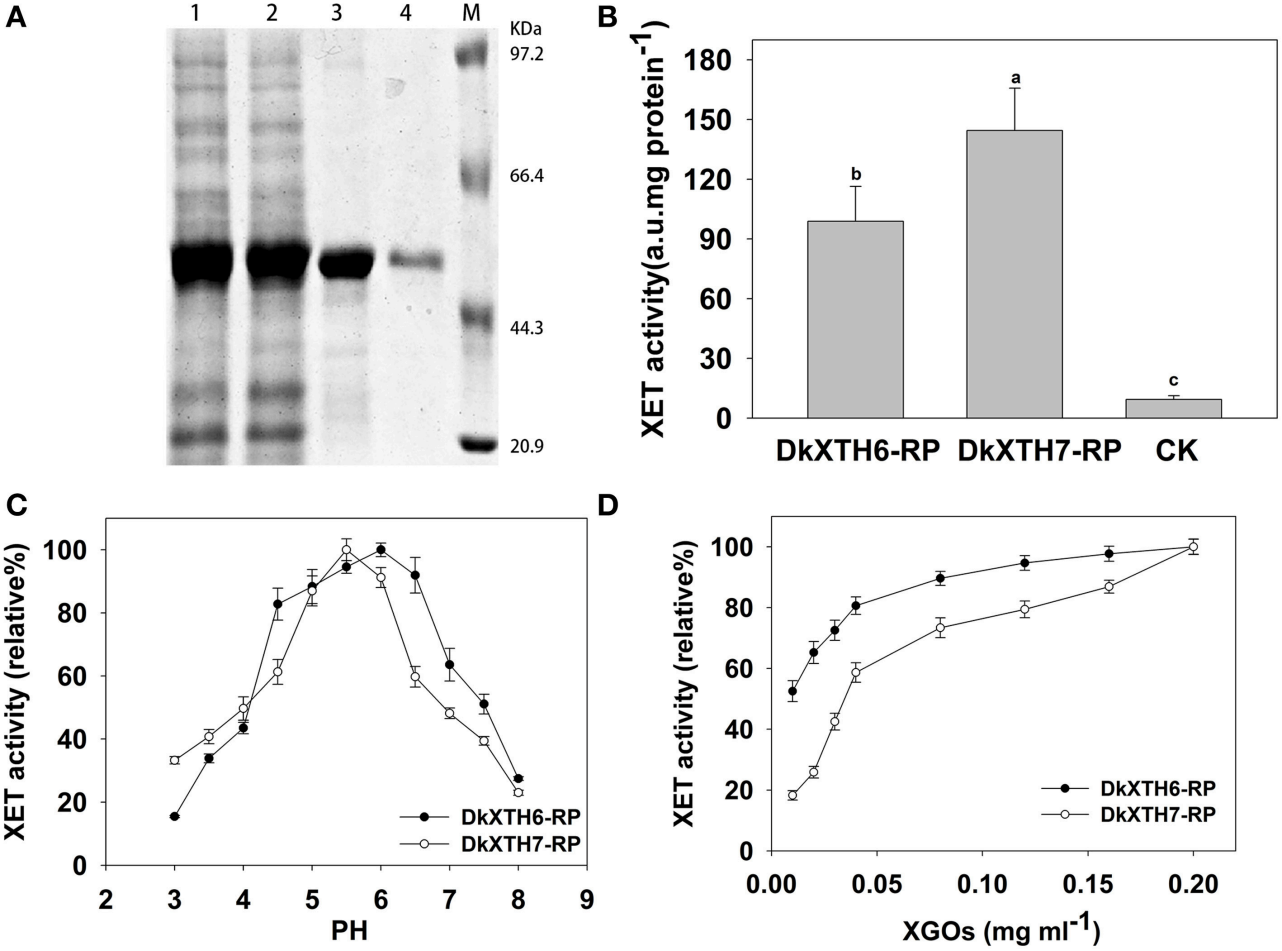
**Expression and activity of recombinant XTH proteins**. **(A)** Proteins were separated on SDS–polyacrylamide gels and stained with Coomassie Blue. Lane 1, total protein (DkXTH6); lane 2, total protein (DkXTH7); lane 3, purified protein (DkXTH6); lane 4, purified protein (DkXTH7); and M, protein marks (Takara, Dalian, China). **(B)** *In vitro* XET assay of recombinant XTH proteins. The XET assay was performed by colorimetric method as described in Section Production and Purification of Recombinant XTH Proteins and Enzyme Activity Analysis. The empty vector pET32a (+) was used as the control. **(C)** The pH–rate profile of recombinant XTH proteins. **(D)** Dependence of XET activity of proteins on the concentration of XGOs. Vertical bars indicate standard errors of three replicates.

To analyze the enzymatic properties of *DkXTH6*- and *DkXTH7*-encoded isoenzymes, recombinant XTH proteins (DkXTH6-RP and DkXTH7-RP) were obtained using prokaryotic expression. The crude proteins of DkXTH6-RP and DkXTH7-RP appeared mostly in the insoluble fraction. The recombined proteins were dissolved in 8 M urea buffer and purified using a Ni-NTA resin column and then were refolded using a reverse urea gradient (Figure 7A). Subsequently, the XET activity of DkXTH6-RP and DkXTH7-RP was measured by a colorimetric assay. Compared with the blank control, both DkXTH6-RP and DkXTH7-RP exhibited remarkably high XET activity (Figure 7B), indicating that the purified recombined proteins were active enzymes. The XEH activity of recombined proteins was also investigated by a viscometric assay, and *T. reesei* cellulase, which could depolymerize xyloglucan by hydrolysis activity, was used as a positive control. After treating xyloglucan with the recombined proteins for a set time, no evident decrease in viscosity of xyloglucan was observed (data not shown), suggesting that both DkXTH6-RP and DkXTH7-RP showed no XEH activity.

Upon testing the pH rate profile of DkXTH6-RP and DkXTH7-RP, two bell-shaped pH profiles were discovered over the pH range of 3–8 (Figure 7C). Meanwhile, the XET activity of both DkXTH6-RP and DkXTH7-RP exhibited an obvious decrease when the pH dropped from 5 to 4, a key feature of XET enzymes (Kallas et al., 2005). In addition, DkXTH6-RP exhibited higher activity when the pH interval was between 4.5 and 6.5, while DkXTH7-RP had a relatively narrow pH optimum of from 5 to 6.

To measure the dependence of the relative XET activity of DkXTH6-RP and DkXTH7-RP on oligosaccharides, different concentrations of XGOs were added as shown in Figure 7D. The relative XET activity dropped at the lower concentration of XGOs, especially when it was below 0.04 mg mL^−1^. However, the relative XET activity of DkXTH6-RP was apparently higher than that in DkXTH7-RP under a low concentration of XGOs. When using 0.01 mg mL^−1^ XGOs, the relative XET activity of DkXTH6-RP was 52.5%, significantly higher than that of DkXTH7-RP (18.3%; *P* < 0.05).

## Discussion

XTHs are encoded by a large multigene family (Rose et al., 2002; Eklof and Brumer, 2010); XTHs individually show various expression patterns and have different responses to hormone and environmental conditions, as reported in fruits such as tomato (Chen et al., 2002; Miedes and Lorences, 2009; Munoz-Bertomeu et al., 2013), apple (Goulao et al., 2007; Munoz-Bertomeu et al., 2013), kiwi (Schroder et al., 1998; Atkinson et al., 2009), and strawberry (Opazo et al., 2010; Concha et al., 2013). Meanwhile, isoenzymes of XTHs exhibit diverse enzymatic properties (Steele and Fry, 2000; Tabuchi et al., 2001), which may confer on them unique roles in cell wall modification (Sulova et al., 2003; Eklof and Brumer, 2010). In persimmon, only five *XTH* genes have been isolated (Han et al., 2015); cloning more these genes and studying the enzymatic properties of the individual isoenzyme could lead to a greater understanding of the roles of specific genes in fruit softening. In this study, two novel *XTH* genes were isolated from persimmon fruit; *DkXTH6* and *DkXTH7* (Table 1). The phylogenetic analysis revealed that DkXTH6 and DkXTH7 were classified along with strict XET enzymes PttXET16A (Baumann et al., 2007) and AdXTH5 (Atkinson et al., 2009), respectively (Figure 1). Sequence analysis indicated that both DkXTH6 and DkXTH7 had the conserved DEIDFEFLG motif (Figure 2A), which is the catalytic domain of XTH (Rose et al., 2002). It has been reported that the length of loop 2 in three-dimensional structures of PttXET16A and TmNXG1 can balance the XET and XEH activities of the enzymes (Mark et al., 2009; Eklof and Brumer, 2010). When we tested the predicted three-dimensional structures of DkXTH6 and DkXTH7, we found that the loop 2 of both peptides had five amino acids like PttXET16A, but less than the 10 amino acids in the loop 2 of TmNXG1 (Figures 2B,C), suggesting that the enzymes encoded by *DkXTH6* and *DkXTH7* may exhibit XET activity rather than XEH activity.

The fruit postharvest softening process is regulated by various genetic factors and biochemical pathways (Giovannoni, 2004). As is well known, persimmon is a typical climacteric fruit, and its softening is regulated primarily by ethylene after harvest (Nakano et al., 2003; Lv et al., 2014). During persimmon fruit postharvest softening, expression of *DkXTH6* and *DkXTH7* followed two opposing patterns. Exogenous propylene and ABA treatment accelerated ethylene production and effectively stimulated the expression level of *DkXTH6*, which seemed to parallel the fruit softening rate (Figures 3, 5). In contrast, exogenous GA_3_ and cold treatment suppressed ethylene production and *DkXTH6* expression, and effectively delayed fruit softening. Similar results have been reported in tomato *SlXTH5* and *SlXTH8* (Munoz-Bertomeu et al., 2013), apple *MdXTH10* and *MdXTH11* (Munoz-Bertomeu et al., 2013), and cherimoya *AcXET1-3* (Li et al., 2009). When testing for *DkXTH7*, the higher expression levels were observed in GA_3_ and cooling fruit, which showed higher firmness. In the case of the strawberry, the expression of *FaXTH1* was typically higher in firmer cultivars than that in softer cultivars, which contributed to cell wall strengthening (Nardi et al., 2014). These results suggested that both *DkXTH6* and *DkXTH7* played important and potentially opposing roles in persimmon fruit softening during storage. *DkXTH6* could be involved in inducing fruit softening; however, *DkXTH7* is associated with fruit firmness containing.

It is worth pointing out that the expression level of *DkXTH6* is higher in mature tissues than fast growing tissues. The opposite is true for *DkXTH7*, which has shown higher expression levels in fast growing tissues. In fast growing tomato fruit, a high level of XET activity was observed, and the expression was highest for the *SlXTH1* gene, which was demonstrated to be involved in cell wall expansion (Ohba et al., 2011). In addition, the coding proteins of *DkXTH6* can be localized in the cell wall by their signal peptide, in contrast to *DkXTH7* proteins, which contained no signal peptide (Figure 6). The overproduction of *Populus euphratica* XTH, a protein localized to the endoplasmic reticulum and cell wall, can cause anatomical and physiological alterations in transgenic tobacco (Han et al., 2013). In maize, the *ZmXTH1* gene, which was demonstrated to be involved in affecting cell wall structure and composition, was weakly bound to cell wall (Genovesi et al., 2008). Taken together, we suggest that DkXTH6 and DkXTH7 may play different roles in cell wall modification.

To date, the enzymatic properties of some XTH isoenzymes have been investigated using recombinant proteins gained from yeast or *E. coli* (Catala et al., 2000; Steele et al., 2001; Chanliaud et al., 2004; Saladie et al., 2006; Goulao et al., 2008). In this paper, the kinetic properties of recombinant DkXTH6 and DkXTH7 proteins (DkXTH6-RP and DkXTH7-RP) were analyzed to explore their divergent roles in persimmon physiological processes. Both DkXTH6-RP and DkXTH7-RP possessed significant XET activity without any detected XEH activity (Figure 7B), similar to the reported activities of recombined AdXTH5, AdXTH7, and AdXTH14 proteins in kiwi fruit (Atkinson et al., 2009) and recombined SlXTH5 protein in tomatoes (Saladie et al., 2006). It has been reported that XETs, which play different roles in cell wall modification, have different affinities for small acceptor molecules (Thompson et al., 1997; Steele and Fry, 2000; Sulova et al., 2003). In the present study, DkXTH6-RP maintained higher relative XET activity than that of DkXTH7-RP at low concentrations of XGOs (Figure 7D), suggesting that DkXTH6-RP had a higher affinity for small acceptor molecules (XGOs). In cultured rose cells, the XET isoenzymes responsible for cell wall restructuring have been demonstrated to have a higher affinity for small acceptor molecules than those involved in cell wall assembly (Thompson et al., 1997; Steele and Fry, 2000). Thus, we supposed that the XTH isoenzymes encoded by *DkXTH6*, which shown higher affinity for small acceptor molecules, are likely to be involved in cell wall restructuring and to play important roles in cell wall structural changes during fruit postharvest softening. This viewpoint is consistent with the report that the XET isoenzymes, which play important roles in the restructuring of existing wall material in sprouting mung bean seedlings, had a higher affinity for small acceptor molecules than that involved in wall assembly by integration of new xyloglucan into the walls in cauliflower florets (Steele and Fry, 2000). By contrast, the isoenzymes encoded by *DkXTH7*, which have a lower affinity for small acceptor molecules, are likely to be responsible for cell wall assembly and to play an important role in cell wall synthesis at fast growing tissues. In expanding tomatoes, Kallas et al. (2005) reported that rapid fruit growth was accompanied with substantial cell wall synthesis, and a high level of XET activity was detected in cell elongation region. After harvest, these isoenzymes may be responsible for maintaining persimmon fruit firmness by integrating new xyloglucan into the cell wall, so that its decrease during storage will result in fruit softening. In tomato, it has been demonstrated that *SlXTH1*, which had its highest expression level in young fast growing fruit, was responsible for maintaining the structural integrity of the cell wall and that its decrease during ripening was responsible for fruit softening (Miedes et al., 2010).

It has been known that there are two types of XET; integrational and restructuring XET (Thompson and Fry, 2001). We speculated that the XET activity of DkXTH7-RP is likely to be responsible for cell wall assembly by “integrational” activity that could integrate newly secreted small xyloglucan molecules into the cell wall. By contrast, the XET activity of DkXTH6-RP is likely to be involved in cell wall restructuring by “restructuring” activity and restructured the preformed wall-bound long xyloglucan polymers. In addition, the pH optimum of DkXTH6-RP was between 4.5 and 6.5, while DkXTH7-RP had a relatively narrow pH optimum of from 5 to 6 (Figure 7C). It has been demonstrated that the XET isoenzymes, which had different functions in mung and nasturtium, exhibited varied PH optima (Steele and Fry, 2000; Sulova et al., 2003).

In conclusion, two novel *XTH* genes were identified from persimmon (*DkXTH6* and *DkXTH7*), presenting opposite expression pattern in tissues, during fruit softening and in response to multiple hormonal and environmental treatments. The recombined DkXTH6 protein had a higher affinity for small acceptor molecules than DkXTH7. The results suggested that DkXTH6 is likely to induce persimmon fruit softening by its involvement in preformed cell wall restructuring and loosening. By contrast, DkXTH7 played an important role in immature tissues during rapid growth, as well as in the maintenance of firmness in mature fruit by taking part in the cell wall synthesis.

## Author contributions

YH and JR conceived and designed research. YH and QB conducted experiments. YH and QB analyzed data. KM, YLH, and JS contributed new reagents or analytical tools. YH and QB wrote the manuscript. The work has not been submitted elsewhere for publication, and all the authors listed have approved the manuscript that is enclosed.

### Conflict of interest statement

The authors declare that the research was conducted in the absence of any commercial or financial relationships that could be construed as a potential conflict of interest. The reviewer DM and Handling editor declared their shared affiliation, and the Handling editor states that the process nevertheless met the standards of a fair and objective review.

## Abbreviations

XTH: xyloglucan endotransglycosylase/hydrolase
GA_3_: gibberellic acid
ABA: abscisic acid
XET: xyloglucan endotransglycosylase
XEH: xyloglucan endohydrolase
ORF: open reading frame
RACE: rapid amplification of cDNA ends
PCR: polymerase chain reactions
pI: theoretical isoelectric point
Ni-NTA: nickel-nitrilotriacetic acid
GFP: green fluorescent protein
XGOs: xyloglucan oligosaccharides.

## References

[B1] Atkinson R. G. Johnston S. L. Yauk Y.-K. Sharma N. N. Schroder R. (2009). Analysis of xyloglucan endotransglucosylase/hydrolase (XTH) gene families in kiwifruit and apple. Postharvest Biol. Technol. 51, 149–157. 10.1016/j.postharvbio.2008.06.014

[B2] Baumann M. J. Eklof J. M. Michel G. Kallas A. M. Teeri T. T. Czjzek M.. (2007). Structural evidence for the evolution of xyloglucanase activity from xyloglucan endo-transglycosylases: biological implications for cell wall metabolism. Plant Cell 19, 1947–1963. 10.1105/tpc.107.051391 17557806 PMC1955714

[B3] Brummell D. A. Harpster M. H. (2001). Cell wall metabolism in fruit softening and quality and its manipulation in transgenic plants. Plant Mol. Biol. 47, 311–340. 10.1023/A:1010656104304 11554479

[B4] Campbell P. Braam J. (1999). Xyloglucan endotransglycosylases: diversity of genes, enzymes and potential wall-modifying functions. Trends Plant Sci. 4, 361–366. 10.1016/S1360-1385(99)01468-5 10462769

[B5] Catala C. Rose J. K. C. Bennett A. B. (2000). Auxin-regulated genes encoding cell wall-modifying proteins are expressed during early tomato fruit growth. Plant Physiol. 122, 527–534. 10.1104/pp.122.2.527 10677445 PMC58889

[B6] Chanliaud E. De Silva J. Strongitharm B. Jeronimidis G. Gidley M. J. (2004). Mechanical effects of plant cell wall enzymes on cellulose/xyloglucan composites. Plant J. 38, 27–37. 10.1111/j.1365-313X.2004.02018.x 15053757

[B7] Chen F. Nonogaki H. Bradford K. J. (2002). A gibberellin-regulated xyloglucan endotransglycosylase gene is expressed in the endosperm cap during tomato seed germination. J. Exp. Bot. 53, 215–223. 10.1093/jexbot/53.367.215 11807125

[B8] Concha C. M. Figueroa N. E. Poblete L. A. Onate F. A. Schwab W. Figueroa C. R. (2013). Methyl jasmonate treatment induces changes in fruit ripening by modifying the expression of several ripening genes in *Fragaria chiloensis* fruit. Plant Physiol. Biochem. 70, 433–444. 10.1016/j.plaphy.2013.06.008 23835361

[B9] Cosgrove D. J. (2005). Growth of the plant cell wall. Nat. Rev. Mol. Cell Biol. 6, 850–861. 10.1038/nrm1746 16261190

[B10] Eklof J. M. Brumer H. (2010). The XTH gene family: an update on enzyme structure, function, and phylogeny in xyloglucan remodeling. Plant Physiol. 153, 456–466. 10.1104/pp.110.156844 20421457 PMC2879796

[B11] Feng H.-L. Zhong Y.-X. Xie H. Chen J.-Y. Li J.-G. Lu W.-J. (2008). Differential expression and regulation of longan XET genes in relation to fruit growth. Plant Sci. 174, 32–37. 10.1016/j.plantsci.2007.09.008

[B12] Figueroa C. R. Pimentel P. Gaete-Eastman C. Moya M. Herrera R. Caligari P. D. S. (2008). Softening rate of the chilean strawberry (*Fragaria chiloensis*) fruit reflects the expression of polygalacturonase and pectate lyase genes. Postharvest Biol. Technol. 49, 210–220. 10.1016/j.postharvbio.2008.01.018

[B13] Fonseca S. Monteiro L. Barreiro M. G. Pais M. S. (2005). Expression of genes encoding cell wall modifying enzymes is induced by cold storage and reflects changes in pear fruit texture. J. Exp. Bot. 56, 2029–2036. 10.1093/jxb/eri201 15955791

[B14] Genovesi V. Fornale S. Fry S. C. Ruel K. Ferrer P. Encina A.. (2008). ZmXTH1, a new xyloglucan endotransglucosylase/hydrolase in maize, affects cell wall structure and composition in *Arabidopsis thaliana*. J. Exp. Bot. 59, 875–889. 10.1093/jxb/ern013 18316315

[B15] Giovannoni J. J. (2004). Genetic regulation of fruit development and ripening. Plant Cell 16, S170–S180. 10.1105/tpc.019158 15010516 PMC2643394

[B16] Goulao L. F. Cosgrove D. J. Oliveira C. M. (2008). Cloning, characterisation and expression analyses of cDNA clones encoding cell wall-modifying enzymes isolated from ripe apples. Postharvest Biol. Technol. 48, 37–51. 10.1016/j.postharvbio.2007.09.022

[B17] Goulao L. F. Santos J. de Sousa I. Oliveira C. M. (2007). Patterns of enzymatic activity of cell wall-modifying enzymes during growth and ripening of apples. Postharvest Biol. Technol. 43, 307–318. 10.1016/j.postharvbio.2006.10.002

[B18] Han Y. Wang W. Sun J. Ding M. Zhao R. Deng S.. (2013). *Populus euphratica* XTH overexpression enhances salinity tolerance by the development of leaf succulence in transgenic tobacco plants. J. Exp. Bot. 64, 4225–4238. 10.1093/jxb/ert229 24085577 PMC3808310

[B19] Han Y. Zhu Q. G. Zhang Z. K. Meng K. Hou Y. L. Ban Q. Y.. (2015). Analysis of xyloglucan endotransglycosylase/hydrolase (XTH) genes and diverse roles of isoenzymes during persimmon fruit development and postharvest softening. PLoS ONE 10:e0123668. 10.1371/journal.pone.0123668 25849978 PMC4388718

[B20] Hiwasa K. Nakano R. Hashimoto A. Matsuzaki M. Murayama H. Inaba A.. (2004). European, chinese and japanese pear fruits exhibit differential softening characteristics during ripening. J. Exp. Bot. 55, 2281–2290. 10.1093/jxb/erh250 15333646

[B21] Ishimaru M. Kobayashi S. (2002). Expression of a xyloglucan endo-transglycosylase gene is closely related to grape berry softening. Plant Sci. 162, 621–628. 10.1016/S0168-9452(01)00608-2

[B22] Johansson P. Brumer H. Baumann M. J. Kallas A. M. Henriksson H. Denman S. E.. (2004). Crystal structures of a poplar xyloglucan endotransglycosylase reveal details of transglycosylation acceptor binding. Plant Cell 16, 874–886. 10.1105/tpc.020065 15020748 PMC412862

[B23] Kallas A. M. Piens K. Denman S. E. Henriksson H. Faldt J. Johansson P. (2005). Enzymatic properties of native and deglycosylated hybrid aspen (*Populus tremula x tremuloides*) xyloglucan endotransglycosylase 16A expressed in *Pichia pastoris*. Biochem. J. 390, 105–113. 10.1042/BJ20041749 15804235 PMC1184566

[B24] Li C.-R. Shen W.-B. Lu W.-J. Jiang Y.-M. Xie J.-H. Chen J.-Y. (2009). 1-MCP delayed softening and affected expression of XET and EXP genes in harvested cherimoya fruit. Postharvest Biol. Technol. 52, 254–259. 10.1016/j.postharvbio.2008.12.009

[B25] Livak K. J. Schmittgen T. D. (2001). Analysis of relative gene expression data using real-time quantitative PCR and the 2(-Delta Delta C(T)) method. Methods 25, 402–408. 10.1006/meth.2001.1262 11846609

[B26] Lu W. Wang Y. Jiang Y. Li J. Liu H. Duan X.. (2006). Differential expression of litchi XET genes in relation to fruit growth. Plant Physiol. Biochem. 44, 707–713. 10.1016/j.plaphy.2006.09.020 17079153

[B27] Lv J. Rao J. Zhu Y. Chang X. Hou Y. Zhu Q. (2014). Cloning and expression of lipoxygenase genes and enzyme activity in ripening persimmon fruit in response to GA and ABA treatments. Postharvest Biol. Technol. 92, 54–61. 10.1016/j.postharvbio.2014.01.015

[B28] Mark P. Baumann M. J. Eklof J. M. Gullfot F. Michel G. Kallas A. M.. (2009). Analysis of nasturtium TmNXG1 complexes by crystallography and molecular dynamics provides detailed insight into substrate recognition by family GH16 xyloglucan endo-transglycosylases and endo-hydrolases. Proteins 75, 820–836. 10.1002/prot.22291 19004021

[B29] Matas A. J. Gapper N. E. Chung M.-Y. Giovannoni J. J. Rose J. K. C. (2009). Biology and genetic engineering of fruit maturation for enhanced quality and shelf-life. Curr. Opin. Biotechnol. 20, 197–203. 10.1016/j.copbio.2009.02.015 19339169

[B30] Miedes E. Herbers K. Sonnewald U. Lorences E. P. (2010). Overexpression of a cell wall enzyme reduces xyloglucan depolymerization and softening of transgenic tomato fruits. J. Agric. Food Chem. 58, 5708–5713. 10.1021/jf100242z 20349961

[B31] Miedes E. Lorences E. P. (2009). Xyloglucan endotransglucosylase/hydrolases (XTHs) during tomato fruit growth and ripening. J. Plant Physiol. 166, 489–498. 10.1016/j.jplph.2008.07.003 18789556

[B32] Munoz-Bertomeu J. Miedes E. Lorences E. P. (2013). Expression of xyloglucan endotransglucosylase/hydrolase (XTH) genes and XET activity in ethylene treated apple and tomato fruits. J. Plant Physiol. 170, 1194–1201. 10.1016/j.jplph.2013.03.015 23628624

[B33] Nakano R. Ogura E. Kubo Y. Inaba A. (2003). Ethylene biosynthesis in detached young persimmon fruit is initiated in calyx and modulated by water loss from the fruit. Plant Physiol. 131, 276–286. 10.1104/pp.010462 12529535 PMC166807

[B34] Nardi C. F. Villarreal N. M. Opazo M. C. Martinez G. A. Moya-Leon M. A. Civello P. M. (2014). Expression of FaXTH1 and FaXTH2 genes in strawberry fruit. Cloning of promoter regions and effect of plant growth regulators. Sci. Hortic. 165, 111–122. 10.1016/j.scienta.2013.10.035

[B35] Nishitani K. (1997). The role of endoxyloglucan transferase in the organization of plant cell walls, in International Review of Cytology - A Survey of Cell Biology, Vol. 173, ed Jeon K. W. (San Diego, CA: Elsevier Academic Press Inc), 157–206. 10.1016/s0074-7696(08)62477-8 9127953

[B36] Nishitani K. Tominaga R. (1992). Endoxyloglucan transferase, a novel class of glycosyltransferase that catalyzes transfer of a segment of xyloglucan molecule to another xyloglucan molecule. J. Biol. Chem. 267, 21058–21064. 1400418

[B37] Nishiyama K. Guis M. Rose J. K. C. Kubo Y. Bennett K. A. Lu W.. (2007). Ethylene regulation of fruit softening and cell wall disassembly in Charentais melon. J. Exp. Bot. 58, 1281–1290. 10.1093/jxb/erl283 17308329

[B38] Ohba T. Takahashi S. Asada K. (2011). Alteration of fruit characteristics in transgenic tomatoes with modified expression of a xyloglucan endotransglucosylase/hydrolase gene. Plant Biotechnol. 28, 25–32. 10.5511/plantbiotechnology.10.0922a

[B39] Opazo M. C. Figueroa C. R. Henriquez J. Herrera R. Bruno C. Valenzuela P. D.. (2010). Characterization of two divergent cDNAs encoding xyloglucan endotransglycosylase/hydrolase (XTH) expressed in *Fragaria chiloensis* fruit. Plant Sci. 179, 479–488. 10.1016/j.plantsci.2010.07.018 21802606

[B40] Payasi A. Mishra N. N. Chaves A. L. S. Singh R. (2009). Biochemistry of fruit softening: an overview. Physiol. Mol. Biol. Plants 15, 103–113. 10.1007/s12298-009-0012-z 23572919 PMC3550369

[B41] Rose J. K. C. Braam J. Fry S. C. Nishitani K. (2002). The XTH family of enzymes involved in xyloglucan endotransglucosylation and endohydrolysis: current perspectives and a new unifying nomenclature. Plant Cell Physiol. 43, 1421–1435. 10.1093/pcp/pcf171 12514239

[B42] Saladie M. Rose J. K. Cosgrove D. J. Catala C. (2006). Characterization of a new xyloglucan endotransglucosylase/hydrolase (XTH) from ripening tomato fruit and implications for the diverse modes of enzymic action. Plant J. 47, 282–295. 10.1111/j.1365-313X.2006.02784.x 16774648

[B43] Schroder R. Atkinson R. G. Langenkamper G. Redgwell R. J. (1998). Biochemical and molecular characterisation of xyloglucan endotransglycosylase from ripe kiwifruit. Planta 204, 242–251. 10.1007/s004250050253 9487728

[B44] Steele N. M. Fry S. C. (2000). Differences in catalytic properties between native isoenzymes of xyloglucan endotransglycosylase (XET). Phytochemistry 54, 667–680. 10.1016/S0031-9422(00)00203-X 10975501

[B45] Steele N. M. Sulova Z. Campbell P. Braam J. Farkas V. Fry S. C. (2001). Ten isoenzymes of xyloglucan endotransglycosylase from plant cell walls select and cleave the donor substrate stochastically. Biochem. J. 355, 671–679. 10.1042/bj3550671 11311129 PMC1221782

[B46] Sulova Z. Baran R. Farkas V. (2003). Divergent modes of action on xyloglucan of two isoenzymes of xyloglucan endo-transglycosylase from *Tropaeolum majus*. Plant Physiol. Biochem. 41, 431–437. 10.1016/S0981-9428(03)00050-0

[B47] Tabuchi A. Mori H. Kamisaka S. Hoson T. (2001). A new type of endo-xyloglucan transferase devoted to xyloglucan hydrolysis in the cell wall of azuki bean epicotyls. Plant Cell Physiol. 42, 154–161. 10.1093/pcp/pce016 11230569

[B48] Thompson J. E. Fry S. C. (2001). Restructuring of Wall-Bound Xyloglucan by Transglycosylation in Living Plant Cells [Online]. Available online at: http://onlinelibrary.wiley.com 10.1046/j.1365-313x.2001.01005.x 11359607

[B49] Thompson J. E. Smith R. C. Fry S. C. (1997). Xyloglucan undergoes interpolymeric transglycosylation during binding to the plant cell wall in vivo: evidence from C-13/H-3 dual labelling and isopycnic centrifugation in caesium trifluoroacetate. Biochem. J. 327, 699–708. 10.1042/bj3270699 9581545 PMC1218846

[B50] Vicente A. R. Saladie M. Rose J. K. C. Labavitch J. M. (2007). The linkage between cell wall metabolism and fruit softening: looking to the future. J. Sci. Food Agric. 87, 1435–1448. 10.1002/jsfa.2837

[B51] Wan C. Y. Wilkins T. A. (1994). A modified hot borate method significantly enhances the yield of high-quality rna from cotton (*Gossypium-hirsutum* l). Anal. Biochem. 223, 7–12. 10.1006/abio.1994.1538 7535022

[B52] Zhang Z. Fu R. Huber D. J. Rao J. Chang X. Hu M. (2012). Expression of expansin gene (CDK-Exp3) and its modulation by exogenous gibberellic acid during ripening and softening of persimmon fruit. Hortscience 47, 378–381.

[B53] Zhong Y.-X. Chen J.-Y. Feng H.-L. Kuang J.-F. Xiao R. Ou M. (2008). Expansin and XET genes are differentially expressed during aril breakdown in harvested longan fruit. J. Am. Soc. Hort Sci. 133, 462–467.

[B54] Zhu Q. Zhang Z. Rao J. Huber D. J. Lv J. Hou Y.. (2013). Identification of xyloglucan endotransglucosylase/hydrolase genes (XTHs) and their expression in persimmon fruit as influenced by 1-methylcyclopropene and gibberellic acid during storage at ambient temperature. Food Chem. 138, 471–477. 10.1016/j.foodchem.2012.09.141 23265513

