# Supplementary material for: Isolation and Characterization of Two Persimmon Xyloglucan Endotransglycosylase/Hydrolase (XTH) Genes That Have Divergent Functions in Cell Wall Modification and Fruit Postharvest Softening
Source: Front Plant Sci. 2016 May 11;7:624. doi: 10.3389/fpls.2016.00624 (PMC4863071; doi:10.3389/fpls.2016.00624)
Supplement: Supplementary file 1 [file Table1.DOC]

**Supplementary Table 1 Expression pattern of *DkXTH6* in persimmon fruits during storage**

| Treatment | Storage time (d) | | | | | | | | |
| --- | --- | --- | --- | --- | --- | --- | --- | --- | --- |
|  | 0 | 4 | 8 | 12 | 16 | 20 | 24 | 28 | 32 |
| Propylene | 1.00±  0.416^a^ | 9.44±  2.467^a^ | 4.07±  0.733^b^ | 2.98±  0.297^b^ |  |  |  |  |  |
| ABA | 1.00±  0.416^a^ | 2.20±  0.491^b^ | 7.72±  0.576^a^ | 2.55±  0.412^bc^ | 3.16±  0.365^a^ |  |  |  |  |
| CK | 1.00±  0.416^a^ | 2.09±  0.304^b^ | 2.02±  0.399^c^ | 4.40±  0.122^a^ | 1.37±  0.247^c^ | 3.16±  0.449^a^ |  |  |  |
| GA_3_ | 1.00±  0.416^a^ | 1.56±  0.420^bc^ | 2.52±  0.379^c^ | 2.08±  0.653^c^ | 2.35±  0.321^b^ | 3.02±  0.150^a^ | 1.89±  0.355^b^ | 2.86±  0.516^a^ |  |
| Cooling | 1.00±  0.416^a^ | 0.47±  0.017^c^ | 2.04±  0.162^c^ | 1.42±  0.097^c^ | 1.08±  0.133^c^ | 1.38±  0.159^b^ | 2.31±  0.049^a^ | 1.81±  0.135^b^ | 1.78±  0.181^a^ |
