# Supplementary material for: Isolation and Characterization of Two Persimmon Xyloglucan Endotransglycosylase/Hydrolase (XTH) Genes That Have Divergent Functions in Cell Wall Modification and Fruit Postharvest Softening
Source: Front Plant Sci. 2016 May 11;7:624. doi: 10.3389/fpls.2016.00624 (PMC4863071; doi:10.3389/fpls.2016.00624)
Supplement: Supplementary file 2 [file Table2.DOC]

**Supplementary Table 2 Expression pattern of *DkXTH7* in persimmon fruits during storage**

| Treatment | Storage time (d) | | | | | | | | |
| --- | --- | --- | --- | --- | --- | --- | --- | --- | --- |
|  | 0 | 4 | 8 | 12 | 16 | 20 | 24 | 28 | 32 |
| Propylene | 1.00±0.384^a^ | 0.21±  0.089^c^ | 0.16±  0.054^c^ | 0.01±  0.002^c^ |  |  |  |  |  |
| ABA | 1.00±0.384^a^ | 0.22±  0.034^c^ | 0.01±  0.003^c^ | 0.32±  0.350^c^ | 0.12±  0.100^b^ |  |  |  |  |
| CK | 1.00±0.384^a^ | 0.66±  0.323^c^ | 1.26±  0.334^c^ | 1.25±  0.363^c^ | 2.37±  0.526^b^ | 4.12±  0.463^c^ |  |  |  |
| GA_3_ | 1.00±0.384^a^ | 87.31±13.314^a^ | 109.11±9.876^b^ | 313.11±  53.317^a^ | 246.03±  64.216^a^ | 96.83±  21.139^b^ | 141.57±  18.869^a^ | 155.71±  57.554^a^ |  |
| Cooling | 1.00±0.384^a^ | 44.34±14.714^b^ | 132.20±29.016^a^ | 148.97±  48.361^b^ | 210.69±  52.452^a^ | 200.15±  84.889^a^ | 67.29±  6.524^b^ | 142.35±  24.226^a^ | 180.16±  47.171^a^ |
